# Graduates of the Medical Colleges, 1856

**Published:** 1856-05

**Authors:** 


*Graduates of the Medical Colleges*, 1856.

| | |
|---|---|
| Jefferson Medical College, | 215 |
| University of Pennsylvania, | 140 |
| University of New York, | 98 |
| University of Nashville, | 85 |
| Medical College of South Carolina, | 85 |
| Medical College of Georgia, | 73 |
| University of Louisiana, | 65 |
| St. Louis Medical College, | 50 |
| Rush Medical College, | 42 |
| College of Physicians and Surgeons, | 40 |
| Cleveland Medical College, | 38 |
| Pennsylvania Medical College, | 37 |
| New York Medical College, | 35 |
| University of Michigan, | 30 |
| University of Missouri, | 28 |
| Massachusetts Medical College, | 28 |
| Philadelphia College of Medicine, | 21 |
| Sterling Medical College, | 20 |
| Miami Medical College, | 18 |
| Medical College of Ohio, | 17 |
| Dartmouth Medical College, | 15 |
| Medical College at Yale, | 13 |
| Medical College of Savannah, | 12 |
| Oglethorp Medical College, | 8 |
| Baltimore College of Dental Surgery, | 17 |
| Ohio College of Dental Surgery, | 10 |

—*American Medical Gazette.*

